# Patient and Public Views on Electronic Health Records and Their Uses in the United Kingdom: Cross-Sectional Survey

**DOI:** 10.2196/jmir.2701

**Published:** 2013-08-23

**Authors:** Serena A Luchenski, Julie E Reed, Cicely Marston, Chrysanthi Papoutsi, Azeem Majeed, Derek Bell

**Affiliations:** ^1^NIHR CLAHRC for Northwest London, Imperial College London, Chelsea & Westminster Hospital NHS Foundation TrustLondonUnited Kingdom; ^2^Department of Social and Environmental Health Research, London School of Hygiene and Tropical MedicineLondonUnited Kingdom; ^3^Department of Primary Care & Public Health, Imperial College LondonLondonUnited Kingdom

**Keywords:** electronic health records, patient attitudes, health care delivery, research, policy

## Abstract

**Background:**

The development and implementation of electronic health records (EHRs) remains an international challenge. Better understanding of patient and public attitudes and the factors that influence overall levels of support toward EHRs is needed to inform policy.

**Objective:**

To explore patient and public attitudes toward integrated EHRs used simultaneously for health care provision, planning and policy, and health research.

**Methods:**

Cross-sectional questionnaire survey administered to patients and members of the public who were recruited from a stratified cluster random sample of 8 outpatient clinics of a major teaching hospital and 8 general practices in London (United Kingdom).

**Results:**

5331 patients and members of the public responded to the survey, with 2857 providing complete data for the analysis presented here. There were moderately high levels of support for integrated EHRs used simultaneously for health care provision, planning and policy, and health research (1785/2857, 62.47%), while 27.93% (798/2857) of participants reported being undecided about whether or not they would support EHR use. There were higher levels of support for specific uses of EHRs. Most participants were in favor of EHRs for personal health care provision (2563/2857, 89.71%), with 66.75% (1907/2857) stating that they would prefer their complete, rather than limited, medical history to be included. Of those “undecided” about integrated EHRs, 87.2% (696/798) were nevertheless in favor of sharing their full (373/798, 46.7%) or limited (323/798, 40.5%) records for health provision purposes. There were similar high levels of support for use of EHRs in health services policy and planning (2274/2857, 79.59%) and research (2325/2857, 81.38%), although 59.75% (1707/2857) and 67.10% (1917/2857) of respondents respectively would prefer their personal identifiers to be removed. Multivariable analysis showed levels of overall support for EHRs decreasing with age. Respondents self-identifying as Black British were more likely to report being undecided or unsupportive of national EHRs. Frequent health services users were more likely to report being supportive than undecided.

**Conclusions:**

Despite previous difficulties with National Health Service (NHS) technology projects, patients and the public generally support the development of integrated EHRs for health care provision, planning and policy, and health research. This support, however, varies between social groups and is not unqualified; relevant safeguards must be in place and patients should be guided in their decision-making process, including increased awareness about the benefits of EHRs for secondary uses.

## Introduction

Electronic health records (EHRs) are often heralded as the cornerstone of modern health care provision, although their development and implementation still remains an international challenge [1-6]. In recent years in the United Kingdom, there have been several policy initiatives aiming to alter the technological landscape in the National Health Service (NHS). The initial focus on centralized, top-down national databases, promoted by the National Programme for Information Technology (NPfIT), has now been displaced by the most recent information strategy launched in 2012, “The Power of Information: Putting all of us in control of the health and care information we need” [7]. This document emphasizes information sharing to ensure local EHR systems work seamlessly “across the entire health and social care sector, both within and between organizations” to provide data to multiple stakeholders [7]. In line with this strategic vision, the Department of Health has announced that hospitals should have implemented electronic patient records by 2015, with fully digitized health records being deployed by 2018 across the health care sector [8-10]. In supporting these aims, the Information Governance Review, newly published at the time of writing, recognizes that the duty to share information in the patients’ interests can be as important as the duty to ensure confidentiality, although the recommendations do not extend the use of identifiable data [11].

Within the policy arena, patients and members of the public are often presented as the primary beneficiaries of this technologically-orientated agenda [8-10]. However, their attitudes towards sharing medical information have been studied in a fragmented fashion. The larger part of previous research has focused either on specific EHR systems (eg, Summary Care Record [12]) or on the use of segregated data for specific purposes (eg, research [13] or care improvement [14]). Most people are generally in favor of EHRs and information sharing, but differences exist depending on the intended use, the type of information being shared, and whether health information is anonymized or not [12-22]. As such, public support is not unqualified. A range of concerns have been documented, including privacy, security, control over access, use, and potential misuse of data [12,17,23-25]. Previous research further shows differences in opinion by age, education level, socioeconomic situation and health status [16,17,19,26]. Furthermore, those with long-term conditions appear more supportive of EHRs for personal health benefit as well as for research [12,23,26].

As we progress toward implementing the information strategy, we require a more in-depth understanding of attitudes toward EHRs and the factors that influence overall levels of support. Information flows in health care are often complex and data are used for multiple purposes, as for example at the interface of care and research [27]. For this reason, we should assess patient views about EHRs that acknowledge their use for multiple purposes including health care provision, health services policy and planning, as well as research. Previous research has provided only basic information on sociodemographic variables, and there has been little work on associations between attitudes to EHRs and the experience of patients in health care. People in regular contact with different health services may have encountered difficulties with information sharing between professionals and thus might perceive EHRs as a solution to these communication barriers.

Against this background of policy change within the United Kingdom, this paper surveys patient and public attitudes based on a more complex view of EHRs as systems that may be used for multiple purposes, as well as examining how attitudes differ when considering specific uses, including health care provision, policy and planning, as well as research. The aim of this study is to enhance understanding of patient and public views about the development of universal patient EHRs and their willingness to share their personal records in a national EHR system, by addressing the following questions:

1. What is the level of patient and public support for a national EHR system overall and for what purposes should it be used?
2. What is the relationship between overall support for a national EHR system and the use of EHRs for health care, planning and policy, and health research?
3. How are health, health care use, and sociodemographic characteristics associated with patient and public support for a national EHR system?

## Methods

We conducted a cross-sectional self-complete questionnaire survey using a stratified cluster random sample of patients and members of the public in an area of West London, United Kingdom. Participants were recruited in 8 outpatient waiting areas of a university teaching hospital and the waiting rooms of 8 general practice (GP) surgeries within the hospital catchment area over a 6-week period beginning August 1, 2011. Eligibility criteria for participation were: (1) 18 years or older, (2) first time filling in the survey, and (3) able to understand the information describing the research study. In total, 5331 individuals participated in the survey. Full details of the study protocol are published elsewhere [28]. The study was approved by the London Dulwich Research Ethics Committee (Ref No 10/H0808/96).

Data were collected on patient and public views about a national EHR system and the purposes for which EHRs should be used if such a system existed. The front page of the questionnaire introduced participants to EHRs using the following definition: “If created, your electronic health record would store everything about your health and the health care you receive from your birth until your death. Electronic health records would bring together in one record all of your separate files, whether stored on paper or on a computer, in all of the different locations where you get health care.” The questionnaire made clear that the study concerned detailed EHRs rather than Summary Care Records. The 31-item questionnaire examined various aspects of patient and public views, but here we present the findings relating to the following 4 key questions:

1. If there was a national electronic health records system, would you want your record to be part of it for your own health care? (“Yes, complete record”; “Yes, partial record”; “No”)
2. If there was a national electronic health records system, would you want your record to be part of it for health services planning and policy? (“Yes, name and address present”; “Yes, name and addressed removed”; “No”)
3. If there was a national electronic health records system, would you want your record to be part of it for health research? (“Yes, name and address present”; “Yes, name and addressed removed”; “No”)
4. Overall, are you in favor of the development of a national electronic health records system? (“Yes”; “No”; “Undecided”)

Further questions recorded details of respondents’ health (whether respondent had a long-term condition or not), health care use (personal health care visits in the previous 6 months) and sociodemographic characteristics (birth year, sex, ethnicity, highest education level attained). The full survey instrument is included in Multimedia Appendix 1.

Only respondents providing complete data for the variables of interest were included in the final statistical analysis (N=2857). We first described the study variables including the number and proportion of the analysis sample. To assess the effects of excluding individuals with missing data, we used logistic regression to compare the distribution of responses for each variable between the analysis sample and the missing sample. We examined the proportions of missing data for questions on the final page of the questionnaire compared with questions at the beginning of the questionnaire to assess the effect of questionnaire length on question completion.

We used descriptive analysis to examine our first 2 questions. The proportions of respondents who would support the development of a national EHR system in the United Kingdom and the proportions of respondents who would allow their EHR to be used for their personal health care, health services planning and policy, and health research were calculated. We then examined the correlation between overall support for a national EHR system and views about the three proposed uses of EHRs using chi-square to test for statistical significance.

We also used a multivariable multinomial regression model to examine associations between views about a national EHR system and health, health care use, and sociodemographic characteristics. We tested for multicollinearity between the independent variables and found all VIF (variance inflation factor) scores to be approximately 1, indicating that they were not highly correlated and could thus be combined in multivariable analyses. *P* values and 95% confidence intervals were adjusted for the clustered design of the survey. All analyses were conducted using Stata IC version 9.0.

## Results

### Participants

We recruited 5331 respondents representing 85.50% of all individuals approached. In total, 2857 out of 5331 (53.59%) respondents completed all relevant sections of the questionnaire and were included in the final analysis. There was no significant difference in the rate of completion for questions at the beginning of the questionnaire compared with those at the end, indicating that respondents were able to complete the questionnaire in the time available.

### Study Population

The sociodemographic, health, and health care use characteristics of the sample are shown in Table 1. The sample is relatively young, with a high proportion of women, and with a high level of educational attainment, while it is also ethnically diverse. A larger proportion of respondents were sampled in outpatient clinics rather than in GP surgeries, which is a characteristic of the survey design. Hospital outpatient clinics were busier than GP surgeries and patients attending the hospital had a higher proportion of health problems than those routinely attending GP surgeries. The recruitment time was divided equally between the two settings to ensure that individuals with long-term health conditions participated in the survey. The majority of respondents have at least 1 long-term condition and accordingly the sample population are moderately frequent health care users.

### Support for a National EHR System and for What Purposes

**Figure 1:**
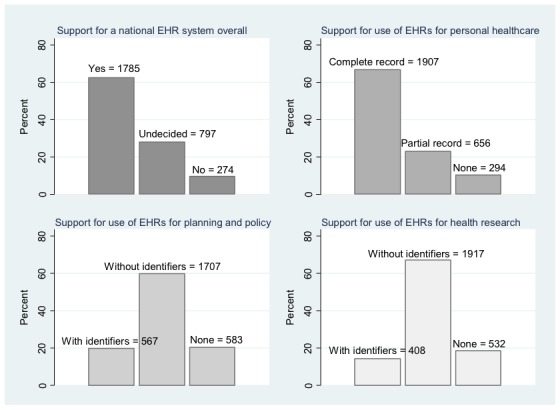
Respondents’ overall preferences for the development of a national electronic health records system and their views on the use of complete or partial records for health care purposes, and identifiable or anonymized records for health services planning and policy, and health research (N=2857).

Respondents’ overall level of support for a national EHR system and the use of EHRs for health care, planning and policy, and health research are presented in Figure 1.

When asked to consider the development of a national EHR system (that would simultaneously support health care, planning and policy, and research), 1785 out of 2857 respondents reported overall support (62.47%), while a large minority of people reported being undecided in their views (n=798, 27.93%). A smaller proportion (n=274, 9.59%) said they would not support a national EHR system used for multiple purposes.

In terms of personal health care provision, responses were more positive with a striking proportion supporting the development of EHRs for this specific purpose (2563 out of 2857, 89.71%). Although 66.75% (n=1907) of respondents would support the use of their complete medical history, almost a quarter of participants (n=656) would allow only limited health information to be part of a national EHR system. 294 out of 2857 (10.29%) said they were opposed to the use of EHRs for health care purposes.

A significant proportion of respondents supported the use of EHRs for planning and policy (n=2274, 79.59%). However, the majority reported that they would only allow their records to be included in an integrated EHR system if personal identifiers had been removed (n=1707, 59.75%). Just one-fifth (n=567, 19.8%) supported the use of identifiable data, with a similar proportion (n=583, 20.4%) opposed to any use of their EHRs for planning and policy.

With regard to using national EHRs for health research, 2325 out of 2857 participants would be similarly supportive of having their records included in the system (81.38%). Yet, only 408 (14.28%) of respondents answered that they would allow their identifiable records to be shared, while 1917 (67.10%) of respondents would prefer having their name and address removed. Almost one-fifth (n=532, 18.62%) said they would not wish their record to be used at all for health research specifically.

**Table 1:** Summary statistics of final analysis sample by sociodemographics, health, and health care use characteristics (N=2857).

Variable		n (%)
**Age category**		
	18-24	226 (7.91)
	25-34 (base)	757 (26.50)
	35-44	614 (21.49)
	45-54	444 (15.54)
	55-64	334 (11.69)
	65-74	294 (10.29)
	75+	188 (6.58)
**Sex**		
	Female (base)	1700 (59.50)
	Male	1157 (40.50)
**Ethnicity**		
	White British (base)	1602 (56.07)
	White Non-British	583 (20.41)
	Black British	207 (7.25)
	Asian British	229 (8.02)
	Mixed	93 (3.26)
	Other	143 (5.01)
**Educational qualifications**		
	None	145 (5.08)
	GCSE	319 (11.17)
	A-Level	288 (10.08)
	Vocational Qualification	335 (11.73)
	Degree	1062 (37.17)
	Higher Degree (base)	708 (24.78)
**Clinic type**		
	GP (base)	953 (33.36)
	Outpatient	1904 (66.64)
**Number of health care visits in the past 6m**		
	0-2 visits (base)	1041 (36.44)
	3-5 visits	998 (34.93)
	6-9 visits	459 (16.07)
	10 plus visits	359 (12.57)
**Long-term conditions**		
	None (base)	1007 (35.25)
	At least one condition	1850 (64.75)

### Relationship Between Overall Support and Support for Specific Purposes

The relationship between individuals’ expressed level of support for a national EHR system and their views about using EHRs for the specific purposes of personal health care, planning and policy, and research are shown in Table 2.

For the 798 (27.93%) of respondents undecided about supporting a national EHR system, the majority (n=696, 87.2%) report that they would support the use of EHRs for their own health care, with 373 out of 798 (46.7%) favoring the use of their complete records and 323 (40.5%) supporting the use of records with limited health information.

Approximately two-thirds of those undecided (n=798) about their overall support for EHRs would support their use for planning and policy (461, 57.77%), and for health research purposes (538, 67.42%), provided the records did not contain personal identifiers.

The majority of those who responded positively (n=1785) to the development of a national EHR system said they would allow their records to be used for health care (1752, 98.15%), planning and policy (1616, 90.53%), and health research (1617, 90.58%). Of those who said they would not be in favor of a national EHR system (n=274), around 40% reported that they would support using EHRs for specific purposes (115 for health care, 101 for planning and policy, and 108 for health research).

### Associations Between Overall Support and Sociodemographics, Health, and Health Care Use

Associations between respondents’ overall level of support for a national EHR system and their sociodemographics, health, and health care use characteristics are shown in Table 3. This multinomial multivariable analysis is interpreted by comparing those who are undecided to those who would support a national EHR system, as well as comparing those who would not be supportive of EHRs to those who expressed positive attitudes. In effect, it is similar to interpreting 2 separate logistic regression models.

There was no clear pattern of association between age and being undecided on support for EHRs overall, or between age and being supportive of such a system. However, there was a graded association between age and lack of support for a national EHR system with older people increasingly more likely to report that they would not be in favor of such a system compared with 25-34 year olds (the largest age category in the sample).

Men were less likely than women to report that they were undecided compared with being positive about EHRs (RR=0.68, 0.59-0.79). Black and Asian British respondents were also more likely to say that they were undecided in their views on EHRs than to say that they would be supportive compared to White British respondents (RR=1.96, 1.34-2.86). Black British respondents were more likely to say they would not support the development of a national EHR system compared with White British respondents (RR=3.72, 2.33-5.94).

Respondents with fewer or no academic qualifications are more likely to report being undecided about their attitudes to EHRs than to report being supportive, compared with those with a higher degree. There are no statistically significant educational differences between people who would support the development of national EHRs and those who would not. There were also no significant differences in this respect between those recruited in GP surgeries and those recruited in hospital outpatient clinics. However, respondents from GP surgeries are more likely to report that they were undecided than positive about national EHRs, compared with those who completed the survey as outpatients in the hospital (RR=1.21, 1.08-1.36).

Individuals who use health services more frequently were less likely to report being undecided about EHRs than to answer that they would be in favor of such a system, compared with less frequent users of health services (0-2 times in the past 6 months). The association is statistically significant for very regular users of health care services (10 or more times in the past 6 months) (RR=0.69, 0.60-0.79). Having a long-term condition was not associated with respondents’ views about a national EHR system.

### Missing Data Analysis

The analysis of missing data in Table 4 shows that those included in the sample have the same age and sex distribution of those not included in the sample. However, respondents with missing data are significantly more likely to be Black (*P*<.001) or Asian (*P*=.02) than White British. Those with lower education levels are also more likely to have missing data than those with a higher degree. The analysis of missing data also shows that the clinical setting did not affect respondents’ likelihood of providing complete data. However, those who have missing data are significantly more likely to use health care services more often and to report no long-term health problems.

Approximately 10% of respondents had missing data on their views about EHRs (ranging from 9.4% to 11.2%), which is lower than for the other analysis variables. However, the analysis showed that those who were excluded from the final analysis sample were significantly more likely to have favorable views towards EHRs for all 4 outcome variables than those who were included (*P*<.001).

**Table 2:** Relationship between overall support for a national EHR system and views about the use of EHRs for personal health care, health services planning and policy, and health research, with Chi square (*χ* ^*2*^ *)* tests used to test for statistical significance (N=2857).

		Support for a national EHR system					
		Yes n (%)	Undecided n (%)	No n (%)	Total n (%)	*χ* ^*2*^	*P*
**Personal health care**							
	Complete record	1484 (83.14)	373 (46.74)	50 (18.25)	1907 (66.75)		
	Partial record	268 (15.01)	323 (40.48)	65 (23.72)	656 (22.96)		
	Neither record	33 (1.84)	102 (12.78)	159 (58.03)	294 (10.29)		
	Total	1785 (100.00)	798 (100.00)	274 (100.00)	2857 (100.00)	1107	<.001
**Health services planning and policy**							
	With identifiers	451 (25.27)	96 (12.03)	20 (7.30)	567 (19.85)		
	Without identifiers	1165 (65.27)	461 (57.77)	81 (29.56)	1707 (59.75)		
	Neither record	169 (9.47)	241 (30.20)	173 (63.14)	583 (20.41)		
	Total	1785 (100.00)	798 (100.00)	274 (100.00)	2857 (100.00)	511	<.001
**Health research**							
	With identifiers	338 (18.94)	62 (7.77)	8 (2.92)	408 (14.28)		
	Without identifiers	1279 (71.65)	538 (67.42)	100 (36.50)	1917 (67.10)		
	Neither record	168 (9.41)	198 (24.81)	166 (60.58)	532 (18.62)		
	Total	1785 (100.00)	798 (100.00)	274 (100.00)	2857 (100.00)	467	<.001

**Table 3:** Relative risks (RR) indicating associations between overall support for a national EHR system and sociodemographic, health, and health care use characteristics. Multinomial logistic regression model comparing those that would support the development of EHRs overall (base=Yes), compared with those who are undecided and those who would not support EHRs; *P* values and 95% CI adjusted for clustering by sampling site (N=2857).

		Overall views on the development of a national EHR system^a^ (base: In favor)							
			Undecided				Against		
Respondent characteristics		Adjusted RR	95% CI		*P* value	Adjusted RR	95% CI	*P* value	
**Age (base: 25-34)**									
	18-24	1.59	(1.13, 2.24)		.008	1.56	(0.83, 2.92)	.17	
	35-44	1.02	(0.79, 1.31)		.90	1.66	(1.17, 2.34)	.004	
	45-54	1.19	(0.94, 1.51)		.14	2.29	(1.39, 3.77)	<.001	
	55-64	1.49	(1.09, 2.03)		.01	2.60	(1.70, 3.98)	<.001	
	65-74	0.84	(0.58, 1.23)		.37	2.53	(1.51, 4.22)	<.001	
	75+	0.97	(0.65, 1.46)		.89	2.86	(1.83, 4.47)	<.001	
**Sex (base: female)**									
	Male	0.68	(0.59, 0.79)		<.001	0.88	(0.67, 1.15)	.36	
**Ethnicity (base: White British)**									
	White non-British	1.14	(0.93, 1.40)		.22	1.00	(0.75, 1.32)	.98	
	Black British	1.96	(1.34, 2.86)		<.001	3.72	(2.33, 5.94)	<.001	
	Asian British	1.43	(1.03, 1.99)		.03	1.37	(0.88, 2.14)	.17	
	Mixed	1.40	(0.97, 2.04)		.08	1.07	(0.55, 2.09)	.85	
	Other	1.23	(0.80, 1.90)		.35	1.18	(0.79 ,1.78)	.42	
**Education (base: higher degree)**									
	None	1.58	(1.03, 2.44)		.04	1.25	(0.60, 2.57)	.55	
	GCSE	1.96	(1.40, 2.75)		<.001	1.27	(0.75, 2.16)	.38	
	A-Level	1.51	(1.08,2.10)		.02	1.00	(0.56, 1.77)	1.00	
	Vocational	1.51	(1.20, 1.90)		<.001	0.85	(0.47, 1.55)	.59	
	Degree	1.29	(1.05, 1.59)		.02	0.93	(0.76, 1.14)	.48	
**Clinic type (base: GP clinic)**									
	Outpatient clinic	1.21	(1.08, 1.36)		<.001	1.13	(0.86, 1.48)	.38	
**Number of health care visits in the past 6 months (base: 0-2 visits)**									
	3-5 visits	0.93	(0.76, 1.15)	.51		0.80	(0.60, 1.05)	.11	
	6-9 visits	0.86	(0.67, 1.09)	.21		0.67	(0.40, 1.12)	.13	
	10 plus visits	0.69	(0.60, 0.79)	<.001		1.21	(0.71, 2.06)	.49	
**Reports long-term medical conditions (base: no conditions)**									
	At least 1 condition	1.21	(0.92, 1.58)	.17		1.35	(0.93, 1.95)	.11	

^a^The questionnaire asked: Overall, are you in favor of the development of a national electronic health records system? (“Yes”; “No”; “Undecided”).

**Table 4:** Support for EHR: Univariable logistic regression of missing data by respondent characteristics (N=5331).

Variable		Missing (%)	Odds ratio	95% CI		*P*	
**Age category (base: 25-34)**		799 (14.99)					
	18-24		0.87		(0.67, 1.15)		.33
	35-44		0.95		(0.81, 1.13)		.56
	45-54		0.90		(0.71, 1.13)		.36
	55-64		0.95		(0.81, 1.13)		.58
	65-74		0.97		(0.77, 1.21)		.78
	75+		0.87		(0.68, 1.11)		.25
**Sex (base: female)**		611 (11.46)					
	Male		1.10		(0.96, 1.27)		.17
**Ethnicity (base: White British)**		1109 (20.80)					
	White non-British		1.14		(1.00, 1.31)		.047
	Black British		0.62		(0.51 ,0.75)		<.001
	Asian British		0.71		(0.53, 0.94)		.02
	Mixed		0.86		(0.68, 1.10)		.23
	Other		0.77		(0.56, 1.05)		.10
**Educational qualifications (base: higher degree)**		833 (15.63)					
	None		0.61		(0.49, 0.75)		<.001
	GCSE		0.90		(0.78, 1.03)		.13
	A-Level		0.76		(0.61, 0.94)		.01
	Vocational qualification		0.87		(0.65, 1.16)		.33
	Degree		0.91		(0.78, 1.06)		.21
**Clinic type (base: GP)**		0 (0)					
	Outpatient		1.01		(0.86, 1.20)		.87
**Number of health care visits in the past 6m (base: 0-2 visits)**		686 (12.87)					
	3-5 visits		1.16		(1.05, 1.29)		.003
	6-9 visits		1.48		(1.27, 1.74)		<.001
	10 plus visits		1.17		(0.97, 1.41)		.10
**Long-term conditions (base: none)**		1103 (20.69)					
	At least 1 condition		0.68		(0.58, 0.81)		<.001
**Overall support for EHRs (base: yes)**		584 (10.95)					
	Undecided		0.67		(0.59, 0.74)		<.001
	No		0.43		(0.36, 0.50)		<.001
**Support for EHRs used for health care purposes (base: complete record)**		499 (9.36)					
	Partial record		0.71		(0.59, 0.84)		<.001
	No		0.43		(0.37, 0.51)		<.001
**Support for EHRs used for health services planning and policy purposes (base: without identifiers)**		566 (10.62)					
	With identifiers		0.78		(0.68, 0.90)		.001
	No		0.56		(0.49, 0.65)		<.001
**Support for EHRs used for health research purposes (base: without identifiers)**		599 (11.24)					
	With identifiers		0.78		(0.68, 0.89)		<.001
	No		0.50		(0.44, 0.58)		<.001

## Discussion

### Principal Findings

This study suggests that there is general support for the development of a national EHR system that would simultaneously use data for multiple purposes, such as personal health care, policy and planning, as well as health research. However, an important minority—about a quarter of participants (n=798, 27.93%)—remain undecided in their views, and nearly 10% (n=274) would be opposed to such a system. When asked about specific purposes for EHRs, over two-thirds of all respondents would support the inclusion of their full medical history and personally identifiable information for personal health care provision. In contrast, for health policy, planning, and research uses, higher support was expressed for use of anonymized EHRs. Even in the group expressing overall negative views towards an integrated EHR system (n=274, 9.59%), there are respondents who would still choose to participate in EHRs if their information was used for specific purposes, such as for their personal health care (n=115, 42.0%), policy and planning (n=101, 36.9%), or health research (n=108, 39.4%). Similarly, over 86% of those undecided (696 out of 798) in their level of support for a national EHR system are supportive of full or partial records being used specifically for their personal health care.

This study also shows significant differences in levels of support depending on sociodemographic characteristics. Age appears to play an important role in support for EHRs with older participants significantly less in favor of EHRs than younger respondents. Black British respondents also show significantly less support than respondents of other ethnic groups. In addition, educational attainment and patterns of health care use differentiate those who report being undecided in their views on EHRs from those who answer that they would be in favor of a national EHR system. However, there is no association between having a long-term condition as measured in this study and support for a national EHR system.

### Strengths and Limitations

This is the first large study to explore patient and public attitudes towards EHRs in the United Kingdom and also the first to draw on a more complex and comprehensive picture of the different potential uses of EHRs, rather than examining their use only for specific purposes. To minimize selection bias, we recruited participants at different days and times following a random sampling design. Although the overall response rate was very high (85.50%), only half of the participants completed the questions for the variables analyzed in this paper (2857 out of 5331, 53.59%). The analysis of missing data shows that there are no age or sex differences between those who were included in the final analysis sample and those who were excluded, but there were ethnicity and education differences. Notably, those with less favorable views were more likely to be excluded from the final analysis. In terms of confounding factors, we measured and adjusted for the main confounding variables in our multivariable analysis; however, the results could still be affected by unmeasured confounders, such as overall levels of trust in the government and authorities. Other methodological considerations related to possible sources of measurement bias are discussed in [28].

### Previous Studies

While other quantitative and qualitative studies have reported that patients and the public would generally support EHRs [12,16] our results contradict previous studies in the United Kingdom and Ireland, which have found higher levels of support in older age groups for information sharing in medical research or GP summary records [17,29]. However, our findings are consistent with similarly large studies in other countries that have found older age groups to be less supportive of EHRs [16]. Our study resonates with previous research showing that ethnic background affects attitudes towards health information sharing: people from BME (Black and Minority Ethnic) communities or people who do not identify themselves as White British have been shown to be less inclined to allow their data to be used for public health and medical research [19,30,31]. In addition, our results on educational differences in opinion between being undecided and being in favor of integrated EHRs extends previous work showing that higher levels of educational attainment are associated with willingness to share health information and support for EHRs [17,29]. Recruitment was carried out in the outpatient and GP population of West London, United Kingdom. Respondents were ethnically diverse with a spectrum of educational backgrounds, which allowed us to sample opinions from a wide range of sociodemographic groups. Overall patterns of opinions may be similar in other areas of London given similarities in sociodemographic and health care characteristics.

### Implications for Research and Policy

The study shows that a proportion of people currently unsupportive or undecided about national EHRs for multiple purposes may nevertheless be amenable to EHRs being used for clearly defined purposes. Patient and public perceptions about inclusion of their records in EHRs for their personal health care mirror levels of overall support for national EHRs, suggesting that considerations of personal health needs might be driving these opinions. Additionally, sociodemographic disparities in levels of support indicate that preferences cannot be considered homogeneous. Introduction of national EHRs may risk widening inequalities for BME groups and the elderly, who are more likely to be against the development of this system. Wider sharing of information may have an effect on their trust toward the health care system and their willingness to seek medical help. Less information on conditions affecting BME and elderly groups may also impact negatively on the potential for health research relevant to these populations and on the planning for services to support their needs. More in-depth research on patient views is needed to draw out the nuances involved in decision-making processes related to wider sharing of health information. Qualitative research studies will enhance our understanding in this area. A more nuanced understanding also has practical relevance in terms of framing policy messages when an EHR is launched and publicized; gaining the support of undecided or opposed groups as well as the public in general could determine whether or not EHRs can be successfully implemented as planned.

Given the well-documented problems inherent in current systems for exchanging patient information between health care professionals and organizations, we hypothesized that the respondents with greater levels of exposure to the health care system would be more acutely aware of the limitations of the current systems and therefore show greater levels of support for EHRs. However, our results in this paper have not indicated a clear relationship between personal health or health care experience and levels of support for EHRs. This suggests that we need to consider how or whether the nuances of health care experience might affect levels of support and use of EHR systems. Understanding an individual’s broader relationship with health care including the need to visit different types of health services, and levels of trust and satisfaction with previous health care encounters may provide greater insight in to the relationship between individuals and their support for EHRs.

### Conclusions

Despite the limited success of the NPfIT program in the United Kingdom, there are high levels of support among patient populations for the establishment of national EHRs. Levels of support are not homogenous and the perspectives of the elderly and Black British populations in particular need to be understood more thoroughly to ensure EHRs do not contribute to widening inequalities in health.

Support is greatest for use of EHRs for personal health care. While support for policy and planning and research is also high, most respondents preferred partial or anonymous data to be used for information sharing rather than complete health records. Our results also suggest that individuals who are currently opposed to, or undecided about the introduction of EHRs for multiple purposes, are nevertheless more likely to be supportive if specific conditions are met regarding the content and purpose of EHRs. Such knowledge can help inform the provision of information for and engagement with specific patient and public groups to ensure that the design of any EHR system is acceptable and effective.

## Multimedia Appendix 1

Survey questionnaire.

## Abbreviations

BME: Black and minority ethnic
CLAHRC: Collaborations for Leadership in Applied Health Research and Care
EHR: electronic health record
GP: general practice
NIHR: National Institute for Health Research
NHS: National Health Service
NPfIT: National Programme for IT
RR: relative risk
